# The needs of parents and their network to influence physical activity and motor development among children 0-to-4-years old: a mixed-methods study protocol

**DOI:** 10.1186/s12889-025-24461-x

**Published:** 2025-10-17

**Authors:** Mary-ann R. Wagijo, Teatske M. Altenburg, Annick Ledebt, Floor Bartels, Robert K. van der Kaap, Katja Bel, Sanne Voorwinden, Corina van Doodewaard, Dave H. H. van Kann, Jessica S. Gubbels, Marleen H. M. de Moor, ASAS-consortium

**Affiliations:** 1 Department of Psychology, Education and Child Studies, Erasmus University Rotterdam, Burgemeester Oudlaan 50, 3062 PA Rotterdam, The Netherlands; 2 Department of Public and Occupational Health, Amsterdam UMC location Vrije Universiteit Amsterdam, Amsterdam, The Netherlands; 3 Amsterdam Public Health, Health Behaviors & Chronic Diseases, Amsterdam, The Netherlands; 4 Department of Human Movement Sciences, Vrije Universiteit Amsterdam, Amsterdam Movement Sciences Research Institute, Institute Brain and Behavior Amsterdam, Amsterdam, The Netherlands; 5 Department of Health Promotion, NUTRIM Institute of Nutrition and Translational Research in Metabolism, Maastricht University, Maastricht, The Netherlands; 6 Research Group Healthy Lifestyle in a Supporting Environment, Centre of Expertise Health Innovation, The Hague University of Applied Sciences, The Hague, The Netherlands; 7 School of Sport Studies, Fontys University of Applied Sciences, Eindhoven, The Netherlands; 8 Research Centre for Movement, School and Sport, Windesheim University of Applied Sciences, Zwolle, the Netherlands

**Keywords:** Physical activity, Motor skills, Early childhood, Family, Parents, Context, System approach, Interviews, Surveys, Experience Sampling Methodology

## Abstract

**Background:**

Promoting physical activity and motor development in early childhood is important for physical and mental health outcomes later in life. However, knowledge on how to influence physical activity and motor development in early life focusing on families and their context in daily life is currently lacking. This study therefore aims to take a systemic approach to investigate the support needs, obstacles and opportunities as perceived by parents and their network to influence physical activity and motor development of young children aged 0 to 4 years.

**Methods:**

This study employs a longitudinal design over a two-month period per family, with mixed methods data collection. Data are collected in 36 families with a child aged 0 to 4 years and a total of 72 network contacts (2 per family). The study is part of the larger Active System for an Active Start project. Extensive qualitative and quantitative data are collected about parents' perceived needs, obstacles and opportunities to influence their children’s physical activity and motor development, as well as information about parental and child characteristics and their social and physical environment. Parents fill out a questionnaire, participate in two interviews at their homes and fill out brief momentary assessments, for two weeks, four times a day, using Experience Sampling Methodology. In addition, the researchers observe parenting behaviour and the home environment during the first interview. Network contacts participate in one interview. Thematic analyses using open and axial group coding will be conducted on the qualitative data. Linear (mixed) modelling will be employed to analyse the quantitative (longitudinal) data.

**Discussion:**

The results of this study will generate in-depth insights into parents’ perceptions and needs to influence their young children’s physical activity and motor development as well as the daily activities of parents and children within their physical and social environments, including the family network. These results will inform the development of intervention and implementation strategies within the larger Active System for an Active Start study, focused on early life and systemic changes to improve physical activity and motor development of young children in the Netherlands.

**Supplementary Information:**

The online version contains supplementary material available at 10.1186/s12889-025-24461-x.

## Background

Early childhood represents a critical period for establishing healthy movement behaviours [1]. Physical activity (PA) in the early years (0 to 4 years) is associated with numerous health benefits, including improved motor skill development, cognitive development, healthy body weight, cardiometabolic, bone and skeletal health and psychological well-being [2–8]. Insufficient PA during early childhood contributes to delayed achievement of motor milestones [9–11], which can be difficult to catch up later in life [9–11].

However, already in infancy a large majority of children (0 to 1 years) do not meet the 24-hour movement guidelines as set by the World Health Organization (WHO)^7^, a trend which continues during toddlerhood and beyond [12–14]. The Dutch PA guidelines recommend for 0 to 1 year old children to be active multiple times a day, with at least 30 minutes of daily PA. For 1- to 4-year-old children the recommendation is to be active for at least 180 minutes per day, including bouts of moderate-to-vigorous PA. It is further recommended that children from both age groups are not ‘secured’ (e.g., strapped in a chair, car seat or bouncer) for longer than 60 minutes at a time, and that they do not sit still for long periods of time [15]. However, most children in the Netherlands between 0 to 4 years do not meet these guidelines [15], since they spend 76–85% of their wake time in sedentary behaviour and only 3–12% engaged in moderate-to-vigorous PA [16, 17].

Different studies and reviews have associated a variety of parental, family system and environmental factors with PA and motor development in children aged 0 to 4 year olds [8, 13, 18–25], which aligns with socio-ecological models of PA [26]. For both PA and motor development, child gender, parental age, family socioeconomic status, education of the parents and family composition have been identified as relevant determinants [25, 27, 28]. Boys seem to be more active compared to girls, having older parents is negatively associated with PA levels and a higher socio-economic status, high education, presence of both parents and having sibling(s) are positively associated with higher PA levels and motor development [21–23, 25, 27, 28].

Parents have great impact on fostering PA and motor development in children from 0 to 4 years old [18, 19]. A quantitative literature review conducted by Hesketh et al. (2017), explored determinants of change in PA in children from 0 to 6 years old and found that maternal role modelling, for example maternal PA, is positively associated with changes in PA levels of children [13]. Parental PA levels have an impact on children's PA, as parents act as role models, positively or negatively influencing their child’s behaviour [19, 20, 22]. Also higher involvement, awareness and co-participation of parents in PA and motor development are associated with positive outcomes [19, 21, 22]. In a study analysing both maternal sports participation and perceived exercise benefits/barriers, only the perceived benefits and barriers were directly associated with children's fitness and motor coordination. Maternal sports participation showed no direct relationship [29]. Furthermore, parents’ perceptions on PA, (work) schedule, health status and preferences of their child are important factors influencing children’s PA and motor development as well [22, 30–32].

The physical and social environments of young children also play a role in their PA and motor development. Physical environmental factors that are positively associated with PA in the early years include the availability of open spaces, playground equipment, and a variety of portable play materials [24]. People within the social environment of the child, such as parents, siblings, and peers, may influence the PA levels and motor development of a child through interactions with the child [33]. In addition, different motor development aspects (for example fine versus gross motor skills) are favoured or impaired, depending on the social cultural context in which a child is raised [27].

Despite the identified determinants, there are still gaps in our knowledge regarding the PA and motor development of 0 to 4 years old children. Firstly, most studies, including intervention studies, are limited to the daycare setting [34, 35]. In the Netherlands, 29% of the children under the age of 4 years does not attend daycare centres [36]. Focusing primarily on the daycare setting limits the reach of children who do not attend daycare as well as of infants in their first months of life, before attending daycare [37]. The home environment of young children is an important setting, but few studies focussed on 0-to-4-year-olds at home. The few studies on home environments reveal an association between developmental stimulation, toys, organization, mother interaction and children nearby in the home environment with PA in infants [38, 39]. Secondly, research on the perception of parents on PA and their needs in daily life to effectively promote PA among 0-to-4-year-old children is still lacking. Research focussing on the context of parents and children is important to develop effective interventions to promote PA in daily life including their home environment. Thirdly, in the social environment of parents and children, different network contacts (such as professionals, e.g., the general practitioner or physiotherapist, and friends or family, e.g., grandparents) can significantly influence health-related behaviours [40–42]. Research on older children (3 to 14 years old) showed that grandparental influence appears to play a significant role in promoting PA [40]. However, similar studies have not yet been conducted among younger children, even though these network contacts are likely also involved in their development as well. Therefore, identifying the perception and needs of network contacts, can provide valuable information to design interventions to influence PA and motor development, while taking the complete context of parents and children into account. In addition, little is known about the view of male caretakers, as they participate less in scientific studies directed at PA-related upbringing of children [20, 43].

This study takes a systemic approach to examine how parents and their informal and formal family networks perceive and support physical activity and motor development in children aged 0 to 4. Specifically, the study aims to address the following research questions:

1. What are the support needs of parents regarding the PA and motor development of their children aged 0 to 4 years and what obstacles and opportunities do they experience in relation to themselves, their child, and the social and physical environment of the family?
2. What daily activities do parents engage in to influence PA and motor development in their young children?
3. How do members of the families’ formal and informal networks perceive the support needs of parents in promoting PA and motor development in young children, and what obstacles and opportunities do they encounter to promote PA and to support parents and children?

## Methods

### Study design

**Fig.1:**
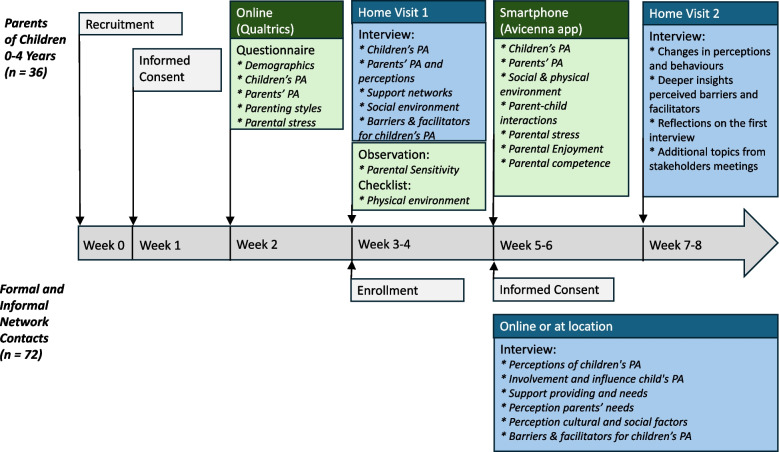
overview of the different research methods and timeline of the study

This study employs a longitudinal design over a two-month period per family, with a mixed methods data collection (see Fig. 1 for an overview of the different research methods and timeline). It is part of the Active System for an Active Start (ASAS) consortium that started in April 2024 and lasts until August 2027. This consortium is a multidisciplinary collaboration between different universities, universities of applied sciences, institutes of secondary vocational education and health-related organizations. The aim is to investigate how PA and motor development among 0-to-4-year-olds can be influenced by employing a systems approach. Data collection of this specific study takes place between September 2024 and November 2025 in three regions geographically spread over the Netherlands: West, North-East and South-East. Ethical approval was granted by the Ethics Committee of Erasmus University Rotterdam (Application number: ETH2324-0902).

### Participants

The study aims to include 36 families (12 families per region) and 72 network contacts (two per family: one contact from their informal network such as a grandparent and one from their formal network such as a daycare professional). To ensure diversity in socioeconomic status of the families, parental gender, ethnicity and achieved motor milestones of the child (walking independently vs. not walking independently), various recruitment strategies were applied. To enhance such a variety in background characteristics, mid-study recruitment evaluations are conducted. Eligibility criteria for parents are: providing care at least 50% of the time for a 0-to-4-year-old child that is born >34 weeks with a birth weight >2500 gram and being proficient in spoken and written Dutch. Eligibility criteria for the network contacts are proficiency in spoken Dutch. Parents can receive a total financial compensation of €45, which they receive stepwise to enhance retention of the parents during the study [44, 45]. Network contacts do not receive financial compensation.

### Recruitment

Recruitment takes place between September 2024 and July 2025 using multiple strategies. In each region, researchers from three universities of applied sciences recruit 12 parents. In the West region, two researchers are involved, in the North-East region three researchers, and in the South-East region also three researchers. A post-doc researcher monitors the overall data collection of the study. Parents will be recruited through social media and local organizations, for example: youth healthcare, community centres, swim schools, playgrounds, and daycare centres. Whenever possible, the researcher will ask employees at these organizations who are already in contact with parents, if they can attend meetings or gatherings to recruit parents in person. When recruiting in person, parents receive an information flyer and verbal information from the researcher. If they are interested in participation, their email address is registered to send them an elaborate information letter as soon as possible. With consent of the parents, their phone number is also registered to enable contact follow-up. If potentially interested parents do not contact the researcher themselves within a week after receiving verbal and/or written information, the researcher will contact them to ask if they are willing to participate or not. If employees from the organizations know parents who are eligible, they can contact the researcher by email or phone to provide the parents’ email address and telephone number (with the parents’ consent to do so). In addition, a digital flyer is posted online on social media (LinkedIn, Instagram and Facebook) and is will be send to the above-mentioned local organizations, with the request to add it in a digital newsletter or other communications with parents. Furthermore, a recruitment poster is designed to hang at local organizations. The digital flyers and posters contain a QR code that parents can scan to register themselves and the researcher will contact them afterwards. To increase trust between parents and researchers, the researcher who collects the parent’s data will be the contact person during the whole process unless this is impossible for practical reasons.

At the end of the first home visit, parents will be asked to identify individuals in their formal networks (e.g., daycare staff, physiotherapists, general practitioners, municipal health employees) and informal networks (e.g., family, friends, neighbours) who are involved in their child’s PA and motor development; examples of networks are provided if needed. One individual form the formal network and one from the informal network will be contacted by the researchers for an interview online or at a location of their choice. Network contacts receive an information letter by email and are contacted after one week to ask if they consent to participate. They will be asked to provide a written informed consent either through email or in person at the start of the interview.

### Data collection procedures

After agreeing to participate, one regional researcher schedules a first home visit with the parent. Parents receive an email confirmation and an online link to a general questionnaire, which they are requested to complete before home visit 1. Home visit 1 includes an interview, a 15-minute observation of parenting behaviour during interaction with their child, a checklist about the physical environment and support to install the Avicenna app on parents’ smartphones for the brief momentary assessments using the Experience Sampling Method (ESM). ESM data collection starts after home visit 1and continues for 14 days. If for any reason, it is not possible to conduct the observation during home visit 1, the observation will take place during home visit 2.

Studies suggest that people’s attitudes and behaviours can shift over time as they reflect on experiences or face new situations and the process of reflection can be more accurately captured with adequate time between data points [46]. Therefore, we chose to schedule the second home visit with the parents after approximately two months. This time frame enhances the chances of capturing both short-term and long-term behavioural or situational changes, increasing the robustness of the data collected, and obtaining a more balanced and complete view of the parents’ experiences and attitudes over time. In addition, the time between the interviews provides the opportunity to identify topics that need to be addressed during home visit 2, based on home visit 1, the network interviews and input gathered from meetings with stakeholders that are part of the broader ASAS study.

### Measurement instruments

#### Questionnaire

The questionnaire (see Table 1 and Supplementary Information) consists of closed-ended questions and optional open-ended responses, collecting the following demographic information: parental gender, age, marital status, ethnicity, education, and employment; household composition (e.g., questions about partner and other children); and child’s age and gender. Collecting demographic data enables researchers to assess and monitor diversity in the sample and allows subgroup analyses [53]. If the participating parent or their partner is not the biological parent of the child, additional questions about the ethnic background of the biological parent are asked. For all other questions about the partner, they refer to the partner they form a household with.

**Table 1:** Overview of study outcomes collected in the questionnaires of the study

	**General Questionnaire**		**ESM Questionnaire**	
	**Study Construct**	**Measurement Instrument**	**Study Construct**	**Measurement Instrument**
Background Characteristics	Gender parent	Self-developed (1 item)		
	Age parent	Self-developed (1 item)		
	Country of birth parent and their biological parents	Self-developed (3 items)		
	Family composition	Self-developed (5 items)		
	Education parent and partner	Self-developed (2 items)		
	Household income & work	Self-developed (3 items)		
	Age child	Self-developed (1 item)		
	Sex child	Self-developed (1 item)		
Child Variables	Physical Activity & Screen time	Movement Behavior Questionnaire- Baby (12 items) or Movement Behavior Questionnaire -Child (18 items) [47]	Momentary Activities	1 item – Adapted from Dunton 2012 [48]
	Organized sport activities	Self-developed (1 item)		
	Daycare during the week	Self-developed (1 item)		
Parent Variables	Physical Activity	International Physical Activity Questionnaire-Short Form (7 items) [49]	Momentary Involvement of parent in activity	Self-developed (1 item)
	Parenting Styles (Authoritative, Authoritarian & Permissive)	Parenting styles & dimensions questionnaire Short Version (32 items) [50]	Momentary Feelings of Enjoyment while involved in activity with child	Self-developed (1 item)
	Parenting Stress	Nijmeegse Ouderlijke Stress Index Short Form (Dutch Short version of the Parenting Stress Index) (33 Items) [51]	Momentary Perceived Parental Stress while involved in activity with child	1 item – Adapted from ESM repository
			Momentary Feelings of Competence while involved in activity with child	4 items – Adapted from de Moor et al. 2023 [52]
Physical Environment			Momentary physical location	3 items - Adapted from Hager 2017 [39]
Social Environment			Momentary presence of other people	1 item – Adapted from Hager 2017^39^

See Supplementary Information for exact phrasing of all question(s) and answer options

Additional sections of the questionnaire assess the children’s participation in organized sports and daycare arrangements, movement behaviours using the validated Movement Behaviour Questionnaire (MBQ-B for children who cannot yet walk, MBQ-C for children who can walk) [47], and parental PA via an instrument developed by the Flemish Institute Healthy Living questionnaire (the Vlaams Instituut voor Gezondheidsbevordering en Ziektepreventie (VIGEZ)), based on the short version of the IPAQ [49]. Parenting styles are assessed using the Parenting Styles and Dimensions Questionnaire [50], and parenting stress using the abbreviated Dutch version of the Parenting Stress Index (the Nijmeegse Ouderlijke Stress Index (NOSI)) [51, 54].

#### Home visit 1

The first home visit includes a semi-structured interview, a checklist of the physical environment and an observation of parenting behaviour during interaction with their child and lasts approximately 1–1.5 hours in total (see Table 2 and Supplementary Information). The interview starts with journey mapping their week planning and further covers the following topics: parental perceptions of children's PA and activities, their own past and current PA levels, their social and physical environment, and barriers and facilitators of promoting their children’s PA. These topics are based on socio-ecological models of PA and previous empirical research, emphasizing the influence of individual, social, and environmental factors on behaviour [22, 26]. Three pilot home visits were conducted before recruitment (without parent-child observation, because the researchers were still in training for this). Based on these pilot visits, question phrasing was refined and additional topics were included in the interview protocol, such as sibling influence and financial considerations for organized PA.

**Table 2:** Overview of Study Topics Collected in the Interviews of the Study

	**Home Interview 1 Parent**	**Home Interview 2 Parent**	**Interview Formal Network**	**Interview Informal Network**
Child (Physical) Activities	Organized activities	Availability toys for child	Role in PA of child	Role in PA of child
	Physical activities the child likes			
	Journey map (Visited places)			
	Toys and activities directed at stimulating PA child			
Parent Physical Activity	Parents’ PA in childhood			
	Current parents’ PA			
Perceptions	Perception parent on character child	Screen time child	Perception physical activity young children	Perception physical activity young children
	Thoughts about PA children		Perception own involvement PA child	Perception own involvement PA child
	Importance PA children		Importance PA children	Importance PA children
	Activities the child is not able to do yet, but the parents would like them to do		Influence own upbringing and culture on PA child	Influence own upbringing and culture on PA child
	Influence own upbringing and culture on PA child		Concerns of parents about PA children	Concerns of parents about PA children
	Description of own role in stimulating PA of their child			
Challenges and Barriers	Concerns about PA or motor development child	Contact with professionals	Providing support general	Providing support general
	Support in their environment	Sources of information about PA and motor development	Providing support PA	Providing support PA
	Impact professional and private life on stimulating PA child	Support family, friends and partner	Needs of parents to stimulate PA	Needs of parents to stimulate PA
			Own needs to stimulate PA children	Own needs to stimulate PA children
Physical Environment	Journey map (weekly activities)			
	Safety neighbourhood			
	Characteristics residence and surrounding			
Social Environment	People involved in physical activities of the child		People involved in physical activities of the child	People involved in physical activities of the child
	Impact of siblings on PA of child			
	Perception of partner on PA of child			
Other	Financial contribution for PA child		Work related activities involving PA children.	
			Tips and tricks for parents	Tips and tricks for parents

The researcher completes a physical environment checklist (see supplementary info 2. Home visit protocol 1 – Parents Guardians) assessing features of the home and neighbourhood environment related to PA and motor development, such as play equipment and outdoor space. Evaluating the home and neighbourhood environment is essential as research suggests that indoor and outdoor play spaces significantly impact children's movement behaviours [55–57].

To assess parenting behaviour in interaction with their child, we employed the OK! [58], which is a structured method to assess parental sensitivity efficiently in real-time in parent-child observations. Parental sensitivity is a construct that stems from attachment theory [59, 60] and is defined as the extent to which a parent correctly interprets and adequately and promptly responds to a child’s signals [61]. Previous research suggests that parental sensitivity is related to the development of young children’s explorative and play behaviours [62]**)**. Researchers complete the OK! App e-learning [58] before the start of data collection to ensure consistent and reliable scoring. The e-learning training includes a user’s self-administered reliability test. In case reliability is insufficient, a second test is taken. If the second test is failed, further use of the OK! app is proscribed, and the researcher would be assigned other tasks. Researchers evaluate parental sensitivity based on predefined criteria in the app yielding a score ranging between 1 (Very strongly insensitive parental behaviour) and 7 (Very strongly sensitive parental behaviour). The observation lasts 15 minutes and utilizes age-appropriate boxes with toys to standardize play interactions. Studies using the OK! observation method have established that a 15-minute timeframe is sufficient for capturing parental sensitivity reliably [58].

#### Experience Sampling Method (ESM)

Daily physical activities of parents and their young children are assessed using ESM via the Avicenna app installed on the phones of parents. ESM is a valid method for studying daily and momentary fluctuations in behaviours, feelings, cognitions, interactions and environments [63, 64]. Parents receive four notifications per day over 14 days to complete very brief questionnaires of 10 items each (approximately 1-2 minutes to complete per notification). The notifications are sent out randomly within four fixed time frames during the day: between 8 and 11 AM, between 11 AM and 2 PM, between 2 and 5 PM and between 5 and 8 PM. Parents are asked to complete the questionnaires within an hour after the notification. Questions address current physical activities, social and physical environments (adapted from Hager et al. and Dunton et al. [39, 48]), parent-child interactions, parental stress, parental enjoyment, and parental competence while interacting with the child (adapted from de Moor 2023 et al. [52]) (see Table 1 and Supplementary Information). The researchers follow up on adherence by checking response rates in the Avicenna Research portal and contact the parents if responses are not received after two days. They remind the parents about the Avicenna app and ask if they need any assistance. A pilot ESM study with eight parents conducted in Spring 2024 (unpublished results) confirmed its feasibility and acceptability. Based on feedback about the answering options and clarity of questions, from the parents through interviews after the pilot, a few items in the ESM survey were adjusted, for example phrased in a different way or an option added in the answers, and the questionnaire was finalized.

#### Informal and formal network interviews

The interviews with parents' formal and informal network contacts (1 each per family) take approximately 45–60 minutes. The interviews are semi-structured and cover the family networks’ perceptions of children's PA and motor development, their involvement and influence regarding PA and motor development of the children, their perception on the influence of PA while they were growing up, their provision of support to parents, the cultural and social impact on PA of children, their perception on support needed by parents, and of barriers and facilitators to influence children’s PA and motor development themselves and through parents.

#### Home visit 2

Approximately two months after home visit 1, a second home visit is scheduled with the parents that lasts approximately 45-60 minutes. This home visit includes a second semi-structured interview that aims to explore possible changes in parental perceptions and behaviours, gain deeper insights into the perceived obstacles and opportunities mentioned by parents in the first interview, and assess reflections on the first interview. To this end, the researcher conducting the interview prepares by reading through and reflecting on the transcript from the first interview before the second interview. Furthermore, new topics are added based on the meetings with stakeholders as part of the broader ASAS study, including trust in PA and health professionals, perception on screen time behaviour, needs for support and equipment availability.

### Data processing and analyses

#### Processing and analyses of interview data

All interviews are transcribed automatically with Amberscript. All generated transcripts are checked manually by the researchers for errors and corrected if needed, to transcribe the interviews at verbatim. After transcription, the interviews will be thematically coded in Atlas.ti [65], using collaborative coding [66]. In each region, one or two researchers will be assigned with the task of coding and participate in a weekly coding meeting where results of the coding process are discussed and aligned over the different regions. In brief, coding will be conducted in three steps: open, axial and selective coding. The identified themes based on the coding will reflect the support needs, obstacles and opportunities of parents and their network to influence PA and motor development of their 0-to-4-year-old children. Twenty percent of the interviews will be independently coded by two other researchers who will not participate in data collection (MRW and FB) and the codebook will be checked by two ASAS consortium members (TMA and JSG). Inter-rater reliability will be computed to assess consistency across coders. Thereafter, the remaining interviews will be double checked on a sample basis. Finally, the interviews will be analyzed with the framework method to identify important patterns, themes and associations [67, 68].

To answer the first research question, about the experienced needs, obstacles and opportunities of parents regarding the PA and motor development of their children aged 0–4 years in relation to themselves, their child, and the social and physical environment of the family, we will examine the interview data of the parents. Themes occurring from the interview data will be thematically presented together with quotes from specific parents to illustrate the themes.

To examine the third research question, about how the families’ formal and informal networks perceive the needs of parents in promoting PA and motor development in young children, and what obstacles and opportunities they encounter, interview data from the network contacts will be analysed and presented using the same procedures as described for the interview data of the parents. 

#### Processing quantitative data

Survey data will be downloaded from Qualtrics and ESM data from Avicenna and saved in SPSS format. Observation data will be manually entered into SPSS. Next, these data will be checked for missings and errors (e.g., implausible values for age) and imputed or corrected where appropriate. Summed scores will be computed for questionnaires where appropriate. Last, all quantitative data derived from the interviews, survey, ESM and observations will be merged into one file in SPSS. For all analysis IBM SPSS Statistics, version 28.0 will be used.

#### Analyses of quantitative data

Descriptive analyses will be conducted to examine the distributions of the study variables (means, standard deviations, range, tests of normality for continuous variables, and frequencies for categorical variables) and the associations among study variables (by computing Pearson correlations and conducting t-tests, analysis of variance and chi-square tests depending on the measurement levels of the relevant variables) in the total study population. Furthermore, we will explore differences in distributions and associations by subgroups (i.e., region, gender parent, socio-economic status, age and motor milestones children).

The second research question, about the daily activities that parents engage in with their young children to promote their PA and motor development, will be answered by analysing the ESM data in combination with survey and observation data. Multilevel regression modelling and/or dynamic structural equation modelling will be employed to examine the interrelationships between PA of the children, parental stress, cognitions and emotions, and parent-child interactions across time and as a function of the social and environmental environment they find themselves in from moment to moment and from day to day [69–71].

## Discussion

This study aims to provide a comprehensive understanding of the perceived support needs, obstacles and opportunities of parents and their network contacts to influence of PA and motor development in young children using a systemic approach.

This study has several strengths. First, the study employs a systematic approach focussing on parents, the family system and their social and physical environment and includes not only the perspective of parents but also that of two important persons from their formal and informal network. Second, the study employs a mixed-methods approach combining various types of qualitative and quantitative data. While the sample size is relatively small for a quantitative study with 36 parents and 72 network contacts and a larger sample could enhance generalizability, the in-depth data acquired by integrating multiple data collection methods (interviews, questionnaires, observations and ESM and network interviews) necessitates a more focused participant sample to ensure feasibility. In addition, the methodological triangulation —where key interview topics overlap with constructs assessed via observations, ESM and/or a questionnaire— enhances the validity and reliability of the findings. Third, the study leverages established collaborations with community partners who maintain direct contact with the populations of interest, facilitating translating research findings into practice.

Despite its strengths, the study also comes with some limitations. Selection bias poses a potential risk due to the intensive nature of the study and the various aspects of the data collection process. Some individuals may refrain from participating, because they might consider the research as time consuming. As a result, participating parents may be more intrinsically motivated and particularly interested in the topic. This self-selection could lead to a less heterogenous study population. However, despite intensive data collection the burden on parents is minimized as much as possible. This is achieved by, for example, combining different methods within a single home visit, eliminating travel time, and scheduling visits at a time convenient for the parent. In addition, researchers conduct observations instead of asking parents to self-report, whenever possible. The use of ESM also allows for brief, in-the-moment data collection, which is perceived as less demanding than other more intensive data collection methods such as keeping diaries [64, 70]. Furthermore, financial reimbursement is offered to increase participation rates. To increase the heterogeneity of the population, participants with various demographic backgrounds are recruited actively in addition to solely passive recruitment methods, such as online calls for participation and flyers. Recruiting participants from lower socio-economic backgrounds, ethnic minorities, and male caregivers remains a known challenge [43, 72]. Recruitment efforts emphasize face-to-face engagement, with follow-up occurring shortly after initial contact to maintain participant’s interest. Scheduling of the first interview at short notice is prioritized to minimize dropout.

Due to data collection by multiple researchers collecting data, the study has a risk of interview bias. This was reduced by discussing and reviewing the interview protocols with all the researchers before data collection, pilot testing in every region with presence of the postdoc researcher in every region and weekly meetings to discuss the interviews and possible obstacles. Response bias is inherent to the study’s data collection methods and is addressed through using different question formats and neutral language.

The combined results of this study will generate in-depth insights into the needs of parents and their network to support physical activity and motor development of their young children. Ultimately, the results of this study are expected to yield important insights that will guide the development and implementation of intervention strategies. The final aim, which will be part of activities of the broader ASAS consortium, is to successfully build communities of practice in the Netherlands to anchor these research findings in practice to effectively support parents and their social network to stimulate PA and motor development among their 0-4 years old child(ren) in daily life.

## Supplementary Information

Supplementary Material 1.

## Data Availability

No datasets were generated or analysed during the current study.

## Abbreviations

ASAS: Active System for an Active Start
PA: Physical Activity
MBQ: Movement Behaviour Questionnaire
VIGEZ: Vlaams Instituut voor Gezondheidsbevordering en Ziektepreventie
NOSI: Nijmeegse Ouderlijke Stress Index
ESM: Experience Sampling Method
