# Supplementary material for: The needs of parents and their network to influence physical activity and motor development among children 0-to-4-years old: a mixed-methods study protocol
Source: BMC Public Health. 2025 Oct 17;25:3523. doi: 10.1186/s12889-025-24461-x (PMC12535135; doi:10.1186/s12889-025-24461-x)
Supplement: Supplementary file 1 — Supplementary Material 1. [file 12889_2025_24461_MOESM1_ESM.docx]

**Supplementary Information**

Supplementary Information to article:

Mary-ann R. Wagijo, Teatske M. Altenburg, Annick Ledebt, Floor Bartels, Robert K. van der Kaap, Katja Bel, Sanne Voorwinden, Corina van Doodewaard, Dave H.H. van Kann, Jessica S. Gubbels, Marleen H.M. de Moor (on behalf of the ASAS-consortium).

The needs of parents and their network to influence physical activity and motor development among children 0-to-4-years old: A mixed-methods study protocol

**1. General Questionnaire**

Thank you for participating in the study "Het Jonge Kind MOOI in Beweging". You will receive the general questionnaire for the study. You will complete this questionnaire once. The answers to these questions are supplementary to the interviews that will take place and by asking these questions via a questionnaire we have time to discuss other topics during the interviews. In this questionnaire we ask questions about your background, your child's, your child's exercise activities, your own exercise activities and how you experience your parenthood. The questions in this list are about your youngest child if you have multiple children. Completing the questionnaire takes about 20 minutes. It is easier to complete the questionnaire on a larger screen, for example on your laptop or computer. If you have any questions about this questionnaire you can contact the practice researcher of the university of applied sciences you are in contact with or Mary-ann Wagijo (researcher Erasmus University) via wagijo@essb.eur.nl.

**Questions about background characteristics**

1. What is your name?
   *We only ask for your name to link your questionnaire to the other questionnaires. If we save your questionnaire, we link it to a code and delete your name immediately.*

Name: ……………………………………………………….
Surname: ……………………………………………….

1. How do you identify yourself in terms of gender?
   - Man
   - Woman
   - Non-binary
   - Otherwise, namely:
   - I don't want to say
2. What is your age?

……. Year.

1. What is your marital status?
   - Married/registered partnership
   - Living together
   - In a relationship, but not living together
   - Separated, living apart
   - Single
   - Widow, widower
2. In which country was the child's **biological mother born?**
   - The Netherlands
   - Suriname
   - Netherlands Antilles
   - Turkey
   - Morocco
   - Otherwise, namely:
   - I don't know
3. In which country was the child's **biological father born?**
   - The Netherlands
   - Suriname
   - Netherlands Antilles
   - Turkey
   - Morocco
   - Otherwise, namely:
   - I don't know
4. What is your relationship with your child?
   - Biological parent🡪 Q 9
   - Non-biological parent/guardian🡪 Q 8
   - Otherwise, namely: ………………………🡪 Q 8
5. In which country were you born?
   - The Netherlands
   - Suriname
   - Netherlands Antilles
   - Turkey
   - Morocco
   - Otherwise, namely:
6. Who do you live with?
   *If you have (step)children who live with you part of the time, you indicate that they live with you if they live with you****at least 50% of the time.*** *(Multiple answers are possible)*
   - With my partner🡪 Question 9a
   - With more than 1 child🡪 question 9c
   - With my parent(s)
   - With another adult/other adults

9a. Is your partner the biological parent of your child?
Yes
No🡪 Q 9b.

9b. In which country was your partner born?

- - - The Netherlands
  - Suriname
  - Netherlands Antilles
  - Turkey
  - Morocco
  - Otherwise, namely:

9c. How many children live in your household?

*If you have (step)children who live with you part of the time, please indicate the total number of children who live with you at home* ***at least 50%*** *of the time.*

- - 2 children
  - 3 children
  - More than 3 children, namely: *fill in field*

1. What is your highest completed education?
   - No education
   - Primary education
   - VMBO, lower level HAVO/VWO or MBO 1
   - Upper secondary education HAVO/VWO or MBO 2-4
   - HBO or WO
   - Otherwise, namely:
   - I don't know
   - I don't want to say
2. **partner** 's highest completed education ?
   - No education
   - Primary education
   - VMBO, lower level HAVO/VWO, MBO 1
   - Upper secondary education HAVO/VWO, MBO 2-4
   - HBO or WO
   - Otherwise, namely:
   - I don't know
   - I don't want to say
   - Not applicable
3. What is your family income?
   *If you live with a partner, this is the total amount that you and your partner receive monthly in salary(ies), allowances and benefits.*
   - Less than €1500 net per month
   - €1500-2500 net per month
   - €2500-3000 net per month
   - €3000-4000 net per month
   - €4000 net per month or more
   - I don't know
   - I don't want to say
4. Do you currently have paid work?
   - Yes, (average) number of hours per week: *fill in field*
   - No
5. Does your partner currently have paid employment?
   - Yes, (average) number of hours per week *fill in field*
   - No
   - Not applicable, I don't have a partner

**Questions about your youngest child**

1. What is the gender of your child?
   - Boy
   - Girl
   - Other
2. What is your child's age in years and months?

___ years and ___ months

**youngest child's skills**

1. Can your child walk independently?
   *By independent walking we mean being able to walk independently without holding a hand.*
   - No 🡪questions Block A.
   - Yes, 🡪ask Block B.

**Block A: Your child's skills (For children who cannot yet walk independently – 11 questions)**

1. What can your child already do?
   *Multiple answers possible.*
   - Lifting the head when lying on the stomach🡪 Q 19
   - Rolling from stomach to back independently🡪 Q 20
   - Rolling from back to stomach independently🡪 Q 20
   - Sitting without support for more than three seconds🡪 Q 20
   - Crawling, bottom-sliding or crawling more than 1.5 meters🡪 Q 20
   - Pulling yourself up and standing🡪 Q 20
   - Walking by the hand🡪 Q 20
   - My child can do all of these things🡪 Q 19
   - My child can't do all these things yet
2. This question is about when your child is awake and you put your child on their tummy to play while you play with them.

   A. In the past week, how many times **a day** did you let your child play on their tummy?
   - 0 6
   - 1 7
   - 2 8
   - 3 9
   - 4 10
   - 5

B. How long do you let your child play on his or her tummy **at a time ?**

- - Less than 5 minutes
  - 5 to 10 minutes
  - 10 to 15 minutes
  - 16 to 20 minutes
  - 21 to 25 minutes
  - 26 to 30 minutes
  - More than 30 minutes

1. Thinking about the **past week** , how long have you been **active?** played with your child on a **normal day** ?
   *Active play can include: crawling on the floor with your child, rolling on the floor with your child, playing in a park, crawling, dancing, chasing your child.*
   - 0 minutes per day
   - 1 to 15 minutes per day
   - 15 to 30 minutes per day
   - 30 to 60 minutes per day
   - 1 to 1.5 hours per day
   - 1.5 to 2 hours per day
   - More than 2 hours per day
2. A. If you think about the **past week** , in a **normal** **day** , how often is your child secured in, for example, a Maxi-Cosi , swing, car seat, stroller, high chair, bouncer, baby carrier or baby sling?
   - 0 🡪Q 21 6
   - 1 7
   - 2 8
   - 3 9
   - 4 10
   - 5

B. If your child was in one of these devices, how long was your child in it during the day?

- - Less than 15 minutes per day
  - 15 to 30 minutes per day
  - 30 to 60 minutes per day
  - 1 to 1.5 hours per day
  - 1.5 to 2 hours per day
  - More than 2 hours per day

1. If you think about the **past week** , on a **normal** **day** , how long did your child watch television programs, videos/internet clips or films on the television, computer, smartphone or tablet?
   - 0 minutes per day
   - 1 to 15 minutes per day
   - 15 to 30 minutes per day
   - 30 to 60 minutes per day
   - 1 to 1.5 hours per day
   - 1.5 to 2 hours per day
   - More than 2 hours per day
2. If you think about the **past week** , on a **normal** **day** , how much time did your child spend playing games, looking at photos or video chatting on the computer, smartphone, game console or tablet?
   - 0 minutes per day
   - 1 to 15 minutes per day
   - 15 to 30 minutes per day
   - 30 to 60 minutes per day
   - 1 to 1.5 hours per day
   - 1.5 to 2 hours per day
   - More than 2 hours per day
3. When you think about the **past week** , during a **normal** **night** , how long does your child sleep in total during the night?
   - Less than 6 hours per night
   - 6 to 8 hours per night
   - 8 to 10 hours per night
   - 10 to 12 hours per night
   - 12 to 14 hours per night
   - More than 14 hours per night
4. If you think about the **past week** , on a **normal** **day** , how long does your child sleep **in total** during the day?
   - Less than 1 hour per day
   - 1 to 2 hours per day
   - 2 to 3 hours per day
   - 3 to 4 hours per day
   - More than 4 hours per day

**Block B: Your child's skills (For children who can walk independently – 17 questions)**

1. What can your child already do?
   *Multiple options possible*

- Running
  - Jumping
  - Moving around on a balance bike, bicycle with training wheels or walker
  - Walking for more than 10 minutes without holding a hand
  - My child can do all of these things
  - My child can't do all these things yet

1. Thinking back over the **past week** , on a **normal WEEKDAY** , how much time did your child spend **actively** played?
   *By active play we mean activities such as: walking, running, dancing, climbing, playing with a ball, riding a (running) bike or swimming.*
   - 0 minutes per day🡪 Q 28
   - 1 to 15 minutes per day
   - 15 to 30 minutes per day
   - 30 to 60 minutes per day
   - 1 to 1.5 hours per day
   - 1.5 to 2 hours per day
   - More than 2 hours per day
2. How much of this time was spent doing vigorous exercise such as running, jumping, dancing or cycling?
   *Note: the time given cannot be longer than your previous answer.*
   - 0 minutes per day
   - 1 to 15 minutes per day
   - 15 to 30 minutes per day
   - 30 to 60 minutes per day
   - 1 to 1.5 hours per day
   - 1.5 to 2 hours per day
   - More than 2 hours per day
3. Thinking back over the **past week** , on a **normal WEEKEND day** , how much time did your child spend **actively** played?
   *By active play we mean activities such as: walking, running, dancing, climbing, playing with a ball, riding a (running) bike or swimming.*
   - 0 minutes per day🡪 Q 30
   - 1 to 15 minutes per day
   - 15 to 30 minutes per day
   - 30 to 60 minutes per day
   - 1 to 1.5 hours per day
   - 1.5 to 2 hours per day
   - More than 2 hours per day
4. How much of this time was spent doing vigorous exercise such as running, jumping, dancing or cycling?
   *Note: the time given cannot be longer than your previous answer.*
   - 0 minutes per day
   - 1 to 15 minutes per day
   - 15 to 30 minutes per day
   - 30 to 60 minutes per day
   - 1 to 1.5 hours per day
   - 1.5 to 2 hours per day
   - More than 2 hours per day
5. If you think about the **past week** , on a **normal** **WEEKDAY** , how much time did your child spend watching television programs, videos/internet clips or movies on the television, computer, smartphone or tablet?
   - 0 minutes per day🡪 Q 32
   - 1 to 15 minutes per day
   - 15 to 30 minutes per day
   - 30 to 60 minutes per day
   - 1 to 1.5 hours per day
   - 1.5 to 2 hours per day
   - 2 to 3 hours per day
   - More than 3 hours per day
6. How much of this time did your child watch these television programs, videos/internet clips or watch movies on the television, computer, smartphone or tablet while your child was **standing** ?

*Please note: the time given must be shorter than your previous answer.*

- - 0 minutes per day
  - 1 to 15 minutes per day
  - 15 to 30 minutes per day
  - 30 to 60 minutes per day
  - 1 to 1.5 hours per day
  - 1.5 to 2 hours per day
  - 2 to 3 hours per day
  - More than 3 hours per day

1. If you think about the **past week** , on a **normal** **WEEKEND day** , how long did your child watch television programs, videos/internet clips or films on the television, computer, smartphone or tablet?
   - 0 minutes per day🡪 Q 34
   - 1 to 15 minutes per day
   - 15 to 30 minutes per day
   - 30 to 60 minutes per day
   - 1 to 1.5 hours per day
   - 1.5 to 2 hours per day
   - 2 to 3 hours per day
   - More than 3 hours per day
2. How much of this time did your child watch these (music) videos on the television, computer, smartphone or tablet while your child was **standing** ?

*Please note: the time given cannot be longer than your previous answer.*

- - 0 minutes per day
  - 1 to 15 minutes per day
  - 15 to 30 minutes per day
  - 30 to 60 minutes per day
  - 1 to 1.5 hours per day
  - 1.5 to 2 hours per day
  - 2 to 3 hours per day
  - More than 3 hours per day

1. Thinking about the **past week** , on a **normal WEEKDAY** , how much time did your child spend playing games, looking at photos, or video chatting on the computer, smartphone, game console, or tablet?
   - 0 minutes per day🡪 Q 36
   - 1 to 15 minutes per day
   - 15 to 30 minutes per day
   - 30 to 60 minutes per day
   - 1 to 1.5 hours per day
   - 1.5 to 2 hours per day
   - 2 to 3 hours per day
   - More than 3 hours per day
2. On a typical weekday, how long did your child spend playing games, looking at photos, or video chatting while your child was **standing** ?

*Please note: the time given must be shorter than your previous answer.*

- - 0 minutes per day
  - 1 to 15 minutes per day
  - 15 to 30 minutes per day
  - 30 to 60 minutes per day
  - 1 to 1.5 hours per day
  - 1.5 to 2 hours per day
  - 2 to 3 hours per day
  - More than 3 hours per day

1. Thinking back over the **past week** , on a **typical WEEKEND day** , how much time did your child spend playing games, looking at photos, or video chatting on the computer, smartphone, game console, or tablet?
   - 0 minutes per day🡪 Q 38
   - 1 to 15 minutes per day
   - 15 to 30 minutes per day
   - 30 to 60 minutes per day
   - 1 to 1.5 hours per day
   - 1.5 to 2 hours per day
   - 2 to 3 hours per day
   - More than 3 hours per day
2. On a typical weekend day, how long did your child spend playing games, looking at photos, or video chatting while your child was **standing** ?

*Please note: the time given cannot be longer than your previous answer.*

- - 0 minutes per day
  - 1 to 15 minutes per day
  - 15 to 30 minutes per day
  - 30 to 60 minutes per day
  - 1 to 1.5 hours per day
  - 1.5 to 2 hours per day
  - 2 to 3 hours per day
  - More than 3 hours per day

1. When you think about the **past week** , during a **normal** **night** , how long does your child sleep in total during the night?
   - Less than 6 hours per night
   - 6 to 8 hours per night
   - 8 to 10 hours per night
   - 10 to 12 hours per night
   - 12 to 14 hours per night
   - More than 14 hours per night
2. If you think about the **past week** , on a **normal** **day** , how long does your child sleep in total during the day?
   - 0 hours per day
   - Less than 1 hour per day
   - 1 to 2 hours per day
   - 2 to 3 hours per day
   - 3 to 4 hours per day
   - More than 4 hours per day
3. In a **normal week** , how many nights does your child have a regular bedtime routine (e.g., bath/shower, reading story)?
   - Never
   - 1 to 2 evenings per week
   - 3 to 4 evenings per week
   - 5 to 6 evenings per week
   - Every evening

**Questions about your child's activities**

1. Does your child participate in an organized sports activity?
   *For example: baby swimming, toddler gymnastics or sports lessons.*
   - Yes 🡪Question 40a and 40b.
   - No 🡪Question 41.

40a. How many days per week does your child participate in a sporting activity?
_____ days per week

40b. How many minutes per week does your child play sports in total per week?
_____ minutes

1. In a **normal week** , how often is your child cared for by:
   *(multiple answers possible)* :
   - A daycare center

___ days per week, ___ hours per week

- - A host parent

___ days per week, ___ hours per week

- - Family

___ days per week, ___ hours per week

- - Other childcare/nanny
    ___ days per week, ___ hours per week
  - I do not use permanent childcare

**Questions about your exercise activities** *(Based on IPAQ short and VIGEZ – 7 questions)*

1. On how many days during a normal week do you walk or run for at least 10 minutes at a time?
   *This includes walking around at work and at home, taking steps to get from place to place, and any other walking you do for recreation, sport, exercise, or leisure.*
   - 0 🡪Q 46 4
   - 1 5
   - 2 6
   - 3 7
2. How much do you usually spend walking or hiking on such a day?
   - _____ hours and _____ minutes per day
   - I don't know
3. On how many days during a normal week do you do activities that require **moderate physical exertion** such as carrying light loads, cycling at a normal pace, light sports such as doubles in tennis, ...? Walking or hiking is not included in these activities.
   - 0 🡪Q48 4
   - 1 5
   - 2 6
   - 3 7
4. **moderate** physical activities
   on such a day ?
   - _____ hours and _____ minutes per day
   - I don't know
5. On how many days during a normal week do you do activities that require heavy physical exertion such as heavy lifting, digging, aerobics, jogging, sports, etc.?
   - 0 🡪Q 50 4
   - 1 5
   - 2 6
   - 3 7
6. How much time do you usually spend on such a day doing **heavy** physical activities?
   - _____ hours and _____ minutes per day
   - I don't know
7. How much time do you spend **sitting** on **weekdays** during a normal week?
   Also consider the moments you spend sitting at your desk at work, at home, watching television or reading, …
   - _____ hours and _____ minutes per day
   - I don't know

If some questions are not yet applicable because your child is too young, please complete the questions with an eye to the future. How would you respond if your child were older?

| *Parenting styles & dimensions questionnaire  Short Version (Robinson et al. 2001)* | Never | Seldom | Sometimes | Often | Always |
| --- | --- | --- | --- | --- | --- |
| I respond to my child's feelings and needs. |  |  |  |  |  |
| I use physical punishment as a way to discipline my child. |  |  |  |  |  |
| I take my child's wishes into account before asking the child to do something. |  |  |  |  |  |
| When my child asks why (he) or (she) should listen, I say: because I say so or because I am your parent and I want you to. |  |  |  |  |  |
| I explain to my child what I think about the child's good and bad behavior. |  |  |  |  |  |
| I spank when my child is disobedient. |  |  |  |  |  |
| I encourage my child to talk about the child's problems. |  |  |  |  |  |
| I find it difficult to discipline my child. |  |  |  |  |  |
| I encourage my child to express himself freely, even if my child disagrees with me. |  |  |  |  |  |
| I punish by taking away my child's privileges without any explanation. |  |  |  |  |  |
| I emphasize the reasons for rules. |  |  |  |  |  |
| I offer comfort and understanding when my child is upset. |  |  |  |  |  |
| I yell or shout when my child misbehaves. |  |  |  |  |  |
| I give compliments when my child behaves well. |  |  |  |  |  |
| I give in to my child when the child causes a fuss about something. |  |  |  |  |  |
| I explode with anger at my child. |  |  |  |  |  |
| I threaten my child with punishment more often than I actually give it. |  |  |  |  |  |
| When making plans for the family, I take my child's preferences into account. |  |  |  |  |  |
| I grab my child when he/she is disobedient. |  |  |  |  |  |
| I give punishments to my child and **don't** actually carry them out. |  |  |  |  |  |
| I show respect for my child's opinions by encouraging my child to express them. |  |  |  |  |  |
| My child has a say in the family rules at home. |  |  |  |  |  |
| I scold and criticize to improve my child. |  |  |  |  |  |
| I spoil my child. |  |  |  |  |  |
| I give my child reasons why rules should be followed. |  |  |  |  |  |
| I use threats as punishment with little or no justification. |  |  |  |  |  |
| I experience warm and intimate times together with my child. |  |  |  |  |  |
| I punish by putting my child somewhere alone with little or no explanation. |  |  |  |  |  |
| I help my child understand the impact of behavior by encouraging my child to talk about the consequences of his/her own actions |  |  |  |  |  |
| I scold or criticize when my child's behavior does not meet my expectations |  |  |  |  |  |
| I explain the consequences of my child's behavior to him/her. |  |  |  |  |  |
| I hit my child when he misbehaves |  |  |  |  |  |

| *NOSIK – Short form Questionnaire* | Completely disagree | Disagree | Disagree/ Disagree | Agree | Totally agree |
| --- | --- | --- | --- | --- | --- |
| de Brock, A. J. L. L., Vermulst, A. A., Gerris, J. R. M., & Abidin, R. R. (1992). *NOSI—Nijmeegse Ouderlijke Stress Index, Handleiding experimentele versie* [NOSI—Nijmegen Parenting Stress Index, Manual experimental version]. Lisse, Netherlands: Swets en Zeitlinger. | | | | | |

# Home Visit Protocol 1 - Parents/guardians

**Information**

**Name of researcher:**

**Date:
Time:
Location:**

**Participant's name:
Child's name:
Other attendees:**

***General introduction***

*Indicate that recording of the conversation is about to start. Introductions. Explanation of what you will do during the home visit. Do you have any questions for me?*

How did you register to participate in this study?

*- Personally approached by researcher.*

*- Via the poster at ….*

*- Via a flyer at ….*

*- Via the website ….*

*- Through the organization….*

*- Through others….*

*- Otherwise, namely….*

**Physical environment**

Journey Map Explanation:
*We have printed out a map of your surroundings and would like to ask questions about your living environment and daily activities.
*if the child is present and old enough you can ask the child to draw a picture of their surroundings or where they like to play in the neighbourhood.*

1. Journey map 🡪print from google maps

Can you describe what a typical day looks like for you and your child?

- *What places do you go to on weekdays? Can you indicate them on the map?*
- *What places do you go to on weekends? Can you indicate them on the map?*
- *How do you get to these places?*
- *Do you feel safe in your neighborhood? Do you feel safe letting your child play outside?*
- *Do you think your neighborhood is suitable for your child to exercise in?*

**Parent perception of exercise**

1. How would you describe your child? And in terms of movement?
2. What do you think of when you think of movement for children aged 0 to 3 years old?
   - *What are the first words that come to mind? Can you give examples of what you mention?*
3. For this research we also developed a definition for exercise:

**Move**

*Any body movement by muscles that requires energy and causes an increased heart rate and breathing. Examples for young children are rolling over, crawling, running, jumping, climbing or playing with a ball. For babies, this also applies: lying on the stomach while the baby is awake and has the space and freedom to move.*
When you hear our definition, what else comes to mind when you think of your child's movement?

- *Can you give examples of this?*

1. How important do you think exercise is for your child?

- *Can you tell us more about this / why is this important to you?*
- *Do you choose toys and outings aimed at getting your child moving?*

1. What exercise activities does (child's name) currently enjoy doing? **(Agard)**- *What activities does (child's name) enjoy doing that involve movement?
   - What activities do you enjoy doing with (child's name) that involve movement and help your child develop his/her motor skills?*
2. Are there certain types of physical activity that you would like (child's name) to be able to do, but cannot yet ? **(Agard)**

- *Explanation: things that the child cannot do because he/she is too young, but also things that he/she cannot do because he/she has less interest or is slower in learning them, for example certain sports or activities to develop motor skills.*
- *Can you explain that a little more?*
- *Why is that important to you?*
- *What do you think it does for your child?*
- *What would it do for you?*

**Social environment**

1. Which people in your environment are involved in stimulating movement and motor skills in your child?

- *How do they encourage your child?*
- *How often and where do they see your child?
  🡪network immediately ask questions*

1. *(Only for parents with multiple children)*What effect do brothers/sisters have on the development of movement of your youngest child? Do you also need other support to stimulate your child's movement because you have multiple/older children? Why or why not?
2. How does your partner feel about your child’s exercise? What kind of support do you get from your partner when it comes to encouraging your child to exercise and what else would you or your partner need?
   *For single parents:* Do you miss the support of a partner in encouraging your child to exercise? Why or why not?
3. Do you miss support from others around you?
4. Many parents indicate that the way they were raised and/or their culture influences the way they raise their children. Also with regard to encouraging exercise. Do you think that plays a role for you too?

- *In what way? Can you explain why/why not?*
- *If partner: Does this (also) play a role for your partner?*
- *Can you give examples of this?*

**Past move older**

1. Can you talk about what physical activities were for you when you were growing up? This could be about any time in your childhood, not just when you were 0-3 years old. **(Agard)**

- *What sports/activities did you do?*
- *Was being active important or not important to your family?*
- *You said that being active (answer previous question) was important to family. Was this true for everyone in your family or just certain people?*

1. What do you do yourself in terms of exercise and sports? **(Agard)**

- *Why don't you do activities? What is the main reason for you to do activities?*

1. We would like to learn whether there are also concerns about the movement of young children and whether parents can be supported more in this. Can you imagine that parents are concerned - and about what?

- *Can you tell us a bit more about that?*
- *Have you ever worried? If so, what, why were you worried?*
- *Where did you look for information? – If not, if you were concerned, what would you do to find information? Where, from whom, and how?*

1. Have you ever received information about stimulating your child to exercise? Was this information easy to understand? In what form and in what way would you like to receive information?

**Parental role in exercise and obstacles and opportunities**

1. How would you describe your role in encouraging your child's movement and motor development?

- *In what ways do you encourage exercise?*
- *Who in your family moves most with your child and why?*
- *How do you experience the moments when you move with your child?*
- *How does your child respond to exercise?*

1. How do other factors play a role in this, such as your work, your own activities, household, other children, social activities, your living environment, etc.?

- *Support in work, time management, parental stress?*
- *What factors stimulate your child's movement? What factors hinder your child's movement?*
- *Factors in living environment, living environment (For example: toddler gym, organized activities, play facilities in the neighborhood, other young children in the neighborhood)*

1. Are there other things parents can do to help their child learn motor skills?

***Definition motor development*** *: The process by which a child gains control over his muscles and learns to use them. Examples of motor skills are the ability to grab, walk, jump and throw.*

- *Can you explain that?*
- *Why do you think that?*

1. What do you think about financial contributions for, for example, your child's sports activities?
   - *Are you prepared to make a financial contribution, why or why not?
   - What do you consider reasonable prices for a sports or exercise activity and why?*

**Parent-child observation**

Observe for 10-15 minutes using the OK! app.
Ask the parent if you can watch the child play with them.

Points of interest/additional information:

**Network contact details**

**Informal network:**

Name: ________________________________________________________________________________

Phone number: _____________________________________________________________________

Email address:_________________________________________________________________________

Relation:______________________________________________________________________________

**Formal network:**

Name: ________________________________________________________________________________

Phone number: _____________________________________________________________________

Email address:_________________________________________________________________________

Relation:______________________________________________________________________________

**Avicenna app**

- Have the participant download the Avicenna app
- Have the participant register with the email address we also use for contact
- Let the participant log in
- The ASAS research code is 3842
- Discuss with the participant when he/she wants to start and set that
- Please note the participant's AID code below

**AID code:**

**Checklist**

*The following questions are filled in by the practical researcher himself based on observations. The estimates of dimensions are discussed in advance and tested during trial interviews.*

Type of property:

- Residential house
- Apartment, ground floor
- Apartment, floor _____
- Detached house
- Otherwise, namely: ______________________________________________________

If the apartment is on the first floor, is there a lift?

- Yes
- No

Is there a playground/playground for children within sight or within a short distance?
*Do they live next to a playing field, playground, park or something similar?*

- Yes, with play equipment suitable for 0-3 years
- Yes, with play equipment, but not suitable for 0-3 years
- Yes, without playground equipment
- No

Is there an outdoor space where the child can play?

- No
- Yes, big garden
- Yes, small garden
- Yes, small balcony/terrace
- Yes, large balcony/terrace
- Yes, shared courtyard/terrace/garden

Is the outdoor area suitable/large enough for the child to play there?

- Insufficient (child cannot play there)
- Reasonable (Child can play there, but not move with a ball or the like)
- Sufficient (child can play with a ball, space for a slide/balance bike)

Are there toys/equipment available in the outdoor area of the home to encourage exercise (e.g. ball, swing, slide, (balance) bike, etc.)?

- Yes No

Which of the following items are in the house?

- Box Walker Game Console (PS, Xbox)
- Rocking chair Ipad Television
- Maxicosi Computer  Dining chair
- Stroller Other, namely ________________________________________

1. **ESM Questionnaire**

**ESM Questionnaire**  *You are about to fill out a short questionnaire about the environment, your child's movement behavior, the interaction with your child and how you experienced this. You may fill out the questions based on the situation you were in just before you received this call to fill out the questionnaire.*

*Environment*

1. I was with my child.

- Yes
- No

1. (a) My child and I were… (if yes)

- At home
- Outside
- En route
- Otherwise, namely…

1. (b) I was … (if no)

- At home
- Outside
- En route
- At work
- Otherwise, namely…

1. My child and I were together with… (multiple answers possible)

- Nobody, I was alone with my child.
- One or more other children
- My partner
- Friends, acquaintances or colleagues
- Strangers
- Otherwise, namely….

*Child's exercise behavior in the past hour*

1. My child was… (multiple answers possible)

- To sleep
- Food or drink
- Sitting or lying down
- Looking at a screen (TV, tablet or phone)
- Playing in the house (active)
- Outdoor play (active)
- Walking, cycling or scootering (outside)
- Otherwise, namely…

*Parental behavior in interaction with child*

1. I did this activity together with my child?

- Yes
- No
- Otherwise, namely…

1. During this activity I have my child…

- Let them play themselves
- Encouraged
- Given a compliment
- Something happened
- Otherwise, namely…

*Parental competence and stress*

1. My child and I had fun together.

Score 0 to 100

1. I felt stress when I was with my child.
   Score 0 to 100
2. I was not sure how much attention to give to my child.
   Score 0 to 100
3. I was comfortable with my child.
   Score 0 to 100
4. I felt confident in my role as a parent.
   Score 0 to 100
5. My patience with my child was limited.
   Score 0 to 100

Thanks for filling out!

# Protocol Interview - Network informal

**Facts**

**Name of practice researcher:**

**Date:
Time:
Location:**

**Name of parent and child:
Name of network participant:
Relationship to child/parent:
Other attendees:**

***General introduction***

*Indicate that recording of the conversation is about to start. Introductions. Explanation of what you are going to do during the interview. Do you have any questions for me?*

**Perception network on movement (Informal + Formal)**

1. What do you think of when you think of movement for young children?
   - *What are the first words that come to mind?
   -* For this research we have also developed a definition for movement:

**Movement**
Any body movement by muscles that requires energy and causes an increased heart rate and breathing. Examples for young children are rolling over, crawling, running, jumping, climbing or playing with a ball. For babies this also applies: lying on the stomach while the baby is awake and has the space and freedom to move.

- *When you hear our definition, what else comes to mind when you think of your child's exercise?*
- *Can you give examples of this?*

1. During the conversation with (parent's name) I asked who they see as their informal network when it comes to their child's exercise. Why do you think (parent's name/guardian) gave your name?
   *Question not applicable to employees of (organized) sports activities.*

- *Can you tell us more about that?*

1. What activities do you do with (child's name), what is your role in the life of (child's name) and (parent's name).

- *Do you also do exercise activities with (child's name)?*
*- What kind of activities? How do you experience these activities? Can you tell us more about that?
- If not, is there a specific reason why you don't do this? Would you like to do exercise activities with (child's name)?*

*- Are there other children aged 0-3 years with whom you do exercise activities?*

1. How important do you think exercise is for a child?

- *Can you tell us more about this, why do you think these points are important?*
- *What do you look for when buying toys or choosing outings for a child?*

**Role of network in movement and obstacles and opportunities (Informal + Formal)**

1. How do you try to support (child's name) and his/her parent/caregiver?

- *Can you tell us more about that?*
- *For what reasons do you offer this support?*

1. How do you try to support (child's name) and his/her parent/caregiver in getting their child moving?

- *Have you ever consciously thought about this? Why or why not?*
- *How else do you think you could support the parent of (child's name)?*

1. What are some things parents can do to help their child learn motor skills?

- *Can you explain that?*
- *Why do you think that?*

1. Many parents indicate that the way they were raised and/or their culture influences the way they raise their children. Also with regard to encouraging exercise. Do you think that plays a role for you too?

- *In what way? Can you explain why/why not?*
- *Can you give examples of this?*

1. We would like to know what parents need when it comes to movement and motor development of young children. What do parents (in general, not specifically for parent of (child's name)) need in your opinion?

- *Why do you think that?*
- *Do you have any examples of this?*

1. What would help you to get (Child's name) moving and developing?

- *What factors help you get the child moving?*
- *What factors make it less possible for you to encourage your child to exercise?*

1. We would like to learn whether there are also concerns about the movement of young children and whether parents can be supported more in this. Can you imagine that parents are concerned - and about what?

- *Can you tell us a bit more about that?*
- *Have you ever worried? If so, what, why were you worried?*
- *Where did you look for information? – If not, if you were concerned, what would you do to find information? Where, from whom, and how?*

**Protocol Interview Formal Network**

# Protocol Interview - Network Formal

**Facts**

**Name of researcher:**

**Date:
Time: Location:**

**Name of parent and child:
Name of network participant: Relationship to child/parent: Other attendees:**

***General introduction***

*Indicate that recording of the conversation is about to start. Introductions. Explanation of what you are going to do during the interview. Do you have any questions for me?*

**Perception network on movement (Informal + Formal)**

1. What do you think of when you think of movement of the young child?
   - *What are the first words that come to mind?*
   For this research we have also drawn up a definition for movement:
    **Move**

Any body movement by muscles that requires energy and causes an increased heart rate and breathing. Examples in young children are rolling over, crawling, running, jumping, climbing or playing with a ball. For babies this also applies: lying on the stomach while the baby is awake and has the space and freedom to move.

- *When you hear our definition, what else comes to mind when you think of your child's exercise?*
- *Can you give examples of this?*

1. During the conversation with (parent's name) I asked who he/she sees as an informal network when it comes to their child's exercise. Why do you think (parent's name/guardian) gave your name?
   *Question not applicable to employees of (organized) sports activities.*- *How often and in what way do you come into contact with (child's name) and (parent's name/guardian).*
2. What activities do you do with (child's name), what is your role in the life of (child's name) and (parent's name).

- *Do you also do exercise activities with (child's name)? Or do you offer advice/guidance for exercise for (child's name)?*
*- What kind of activities?*

*- How do you experience these activities? Can you tell us more about them?*

1. How important do you think exercise is for a child in general?

- *Can you tell us more about this, why do you think these points are important?*
- *Do you know what the guidelines are for a child's exercise?*
- *If applicable (e.g. childcare) : do you choose toys and outings aimed at children's movement? If so, what kind of toys and outings? If not, what do you look for when purchasing toys and choosing outings?*

1. When you look at (child's name), which points do you find specifically important for him/her?

- *How does (child's name) movement mainly stimulate?*
- *Can you tell us more about this, what is your reason for doing these specific activities with (child's name)?*

**Exercise and contact with young children (Formal network)**

1. In what ways do you come into contact with young children and deal with their movement and motor skills?

- If a daycare organization: do you organize activities focused on movement? What kind? Can you tell us more about that? How do the children respond to this? How do the parents/guardians respond to this?
- If a (medical) professional: What information/guidance do you provide to parents/caregivers in the area of movement and motor skills development?
- If employee (organized) sports activities: What activities do you do with (child's name)? Can you tell us more about this? How do the children react to this? How do the parents/guardians react to this?

**Role of network in movement and obstacles and opportunities (Informal + Formal)**

1. How do you try to support (child's name) and his/her parent/caregiver?

- *Can you tell us more about that?*
- *For what reasons do you offer this support?*

1. How do you try to support (child's name) and his/her parent/caregiver in getting their child moving?

- *Have you ever consciously thought about this? Why or why not?*
- *How else do you think you could support the parent of (child's name)?*

1. What tips do you give parents to stimulate their child's motor skills and movement?

- *Why these tips?*
- *How do parents respond to this?*

1. Many parents indicate that the way they were raised and/or their culture influences the way they raise their children. Also with regard to encouraging exercise.

- *Do you think that plays a role for you too?*
- *In what way? Can you explain why/why not?*
- *Can you give examples of this?*

1. We would like to know what parents need when it comes to movement and motor development of young children. What do the parents of (child's name) need in your opinion? And what do parents/caregivers need in general?

- *Why do you think that?*
- *Do you have any examples of this?*

1. What would help you to get young children to move and develop well/even more?

- *Which factors help to get the child moving?*
- *What factors make it less possible for you to encourage your child to exercise?*

1. We would like to learn whether there are also concerns about the movement of young children and whether parents can be supported more in this. Can you imagine that parents are concerned - and about what?

- *Can you tell us a bit more about that?*
- *Have you ever worried? If so, what, why were you worried?*

# **Home Visit Protocol 2 - Parents/guardians**

**Facts**

**Name of researcher:**

**Date:
Time:
Location:
Participant's name:
Child's name: Other attendees:**

***General introduction***

*Indicate that recording of the conversation is about to start. Explanation of what you will do during the home visit. Do you have any questions for me?*

**Flashback**

1. How did you experience the first interview?
   - How do you look back on our first conversation?
   - What are the key things you took away from the conversation?
   - And to what extent has this changed your vision/view/idea on the movement of young children?
   - What influence has this had on the child's exercise behavior?
2. **Have you discussed the first interview with people around you, such as your partner/family/friends or professionals?**

- Can you tell us a little more about this?
- Who did you talk to about it?

1. *Some points may already be known from the first conversation. With this question we want to find out where the parents get ideas and inspiration from to keep their child busy at home during a normal week . So not even at “special” moments such as holidays, days off etc.***How do you find inspiration for daily exercise activities for your child at home and in your immediate environment. Are there other activities for which you find inspiration and in what way?**

*Think of inspiration about games and activities that they can do at home, for example via: social media accounts, materials (such as GGD posters with exercise tips or exercise calendars), (play) appointments with other parents, YouTube , Google, friends/family who they ask for information or plan activities with, etc.*

**Topics WP1**

1. *The formulation of this question depends on what came out of the first interview.*
   **What is your perception of screen use by children between 0 and 4 years old?**
   *- At what times and for what reasons do you use a screen for your child? What factors play a role in the decision whether or not to let your child use a screen?
   - What do you think of your child's screen use? Do you actively do anything to limit/encourage this? And how does this compare to other children around you?
   - How would you describe your own screen use?*
2. *Asking for trust in professionals – keep questions open and do not direct, but try to go deeper into trust/distrust depending on the conversation and answers. We do not want to ask directly about trust. Answers may already have been given in the first conversation, but it is possible that participants now feel more confident and will go deeper into this.***What are your experiences with health advice and exercise advice from professionals?**

   - *If the participant does not know which professionals, examples can be given: Think of GGD consultations, GP/paediatrician, midwife, etc.
   - If you want advice about exercise, how do you look for it and from whom would you like to receive advice (professionals/informal network) ? And in what way?*
3. *With this question we want to know whether parents would use a larger range of toys or obtain information if there were central places to rent toys and to gain knowledge about suitable toys. What would stop them or encourage them to use such initiatives, do they need this?*
   **In your opinion, do you have sufficient and suitable toys at home for your child and knowledge about toys to stimulate movement?

   Do you have access to toys through a library or toy library or friends/family?

   If there were a place here to rent toys for your child, why would you or would you not use it?

   In what ways can the use of toys aimed at movement be stimulated among parents in your opinion?**

**Additional Topics**

1. Further explore support needs or ask additional questions – based on points arising from home visit 1.
2. Within our research, various organizations have been brought together that meet every so often. What would you as a parent want them to do/map/research/develop?
3. Is there anything else you wanted to say about your child's movement after the first interview?

**Closure**

1. If interim results are known, a short feedback can be given. Also a feedback of the conversation with their informal and formal network🡪 **PLEASE NOTE:** the conversations with the network are confidential, so no substantive feedback may be provided.
2. Discuss the tips and tricks that parents can use to stimulate their child's movement and motor skills.
3. The project will continue for the next few years. If participants are still needed for activities at a later stage, may the researchers contact you?
4. **Please note the participant's details for compensation payment.**

Name:
IBAN: Address: Postcode: City:
